# Changes in handwashing and hygiene product usage patterns in Korea before and after the outbreak of COVID-19

**DOI:** 10.1186/s12302-021-00517-8

**Published:** 2021-07-03

**Authors:** KeunOh Choi, Seunghye Sim, Junyeong Choi, Choa Park, Yoonhee Uhm, Eunkyung Lim, Augustine Yonghwi Kim, Seong Joon Yoo, YoungJoo Lee

**Affiliations:** 1 Department of Integrated Bioscience and Biotechnology, College of Life Science, Sejong University, Kwangjingu, Kunjadong, Seoul, 05006 Korea; 2 Korea National Council of Consumer Organizations, SEOUL YWCA Bld. #701, 20, Myungdong11gil, Jung-Gu, Seoul, Korea; 3 Department of Food Science and Biotechnology, Sejong University, Kwangjingu, Kunjadong, Seoul, 05006 Korea; 4 Department of Computer Science and Engineering, College of Software Convergence, Sejong University, Kwangjingu, Kunjadong, Seoul, 05006 Korea

**Keywords:** COVID-19, Hand hygiene, Handwashing, Hand sanitizer, Liquid hand soap, Bar soap, Korea

## Abstract

**Background:**

The World Health Organization declared COVID-19, the disease caused by severe acute respiratory syndrome coronavirus-2 (SARS-CoV-2), a global pandemic on March 11, 2020. Non-pharmaceutical interventions such as social distancing, handwashing, using hand sanitizer, and wearing facial masks are recommended as the first line of protection against COVID-19. Encouraging hand hygiene may be one of the most cost-effective means of reducing the global burden of disease.

**Methods:**

This study uses a web-based questionnaire to evaluate the usage patterns and consumer perceptions of the effectiveness and health safety of bar soap, liquid hand soap, and hand sanitizer products before and after the spread of COVID-19.

**Results:**

The results show that since the outbreak of COVID-19, the number of consumers who primarily use bar soap has decreased from 71.8 to 51.4%, the number of those who primarily use liquid hand soap has increased from 23.5 to 41.3%, and the number of those who use and carry hand sanitizer has increased. The frequency of use, duration of use, and amount used of all three products have increased significantly since the COVID-19 outbreak. Finally, consumer perception of the products’ preventive effect against COVID-19 is higher for liquid hand soap and hand sanitizer than it is for bar soap.

**Conclusions:**

Because use of hand sanitizers has increased, public health guidelines must address the potential risks associated them. Our data also show that the public is abiding by the recommendations of the regulatory authorities. As handwashing has become important in preventing COVID-19 infections, the results of our study will support the development of better handwashing guidelines and a public health campaign.

**Supplementary Information:**

The online version contains supplementary material available at 10.1186/s12302-021-00517-8.

## Highlights

Handwashing habits were evaluated before and after the spread of COVID-19.

Handwashing habits increased significantly since the COVID-19 outbreak.

Handwashing guidelines and campaigns be developed informing the potential risks.

**Supplementary Information:**

The online version contains supplementary material available at 10.1186/s12302-021-00517-8.

## Introduction

A number of pneumonia cases, in which the cause was unknown, appeared in Wuhan, Hubei Province, China, in December 2019 [1]. On January 7, the Chinese Center for Disease Control and Prevention (CDC) detected a novel coronavirus in a patient and identified it as the etiologic agent of this cluster of pneumonia cases [2]; the virus was named severe acute respiratory syndrome coronavirus-2 (SARS-CoV-2) [3]. The number of COVID-19 cases outside of China began to surge after February 20: new infections were reported in Italy, Korea, and Japan and rapidly spread to European countries in March [4, 5]. The World Health Organization (WHO) declared COVID-19 a global pandemic on March 11, 2020.

COVID-19 can be transmitted through droplets and contact. It is not yet clear whether COVID-19 can be transmitted through aerosols containing the virus, including absorption of the virus through the digestive tract [6]. Contamination through the respiratory droplets and feces of COVID-19 patients suggests that environmental factors are a potential transmission medium, indicating the need for strict adherence to environmental and hand hygiene guidelines.

With no available drugs or vaccines, non-pharmaceutical interventions such as social distancing, handwashing, using hand sanitizer, and wearing a face mask are recommended as the first line of protection against COVID-19 [7, 8]. A recent report showed that members of the public are compliant with easy, straightforward, and familiar recommendations, such as handwashing, wearing masks or avoiding touching the face [9]. Handwashing is key to reducing the spread and transmission of infectious diseases, such as pneumonia, influenza, HIV-related infections, and environmental enteropathies.

Previous studies have suggested that encouraging hand hygiene may be one of the most cost-effective means of reducing the global burden of disease [10]. However, excessive handwashing may cause various adverse dermatologic effects, such as irritant contact dermatitis or, uncommonly, allergic contact dermatitis [11]. Since February 2020, Google searches for “washing hands” and “hand sanitizer” have increased [12, 13], indicating the need for public information and guidelines related to hand hygiene.

In this study, we analyzed changes in handwashing patterns before and after the pandemic to provide better handwashing guidelines and to prepare for future public health crises. Our research on changes in handwashing behavior could contribute to providing consumers with the appropriate handwashing guidelines and developing better strategies for a public campaign.

## Methods

### Study population

A total of 1000 Korean consumers participated in this study, and the survey was conducted between July 27 and August 19, 2020. Respondents were appropriately distributed by gender and age group (20s, 30s, 40s, 50s, and 60s).

### Data collection and analysis

A web-based questionnaire was used to evaluate the usage patterns and perceptions of bar soap, liquid hand soap, and hand sanitizer before and after the spread of COVID-19. The questionnaire first asked about participants’ handwashing habits, including questions about which products they use, the amount of product they use, the duration of each, if they have any concerns about the products’ safety, and their perception that the product can prevent against COVID-19 infection. To determine handwashing patterns before the COVID-19 pandemic, participants were asked to answer a series of questions based on their habits one month prior to the outbreak. For each product group, questions about participants’ safety concerns and their perception of the product’s ability to prevent the spread of COVID-19 were asked on a 7-point scale. The questionnaire was conducted in Korean, but an English translation is provided in the Additional file 1.

Data were analyzed using RStudio version 1.3.1073 (The R Foundation for Statistical Computing, Vienna, Austria) and SPSS version 21.0 software (SPSS Inc., Chicago, IL, USA). A *p*-value < 0.05 was considered significant.

## Results

### Demographic characteristics of the questionnaire respondents

The demographic breakdown of the questionnaire respondents is 50.9% male and 49.1% female, which is representative of the national population (50.1% male and 49.9% female). We constructed five age groups: 20s (20–29 years), 30s (30–39 years), 40s (40–49 years), 50s (50–59 years), and 60s (60–69 years). These groups contained 19.4%, 19.9%, 23.5%, 24.4%, and 12.8% of the respondents, respectively. The group with the highest bias (percentage of the questionnaire respondents compared with the national population) was the 60s (–2.9%) (Table 1).

**Table 1:** Demographic characteristics of respondents

Groups		Respondents *N* (%)	National statistics *N* (%)
Gender	Male	509 (50.9)	25,877,195 (50.1)
	Female	491 (49.1)	25,752,317 (49.9)
Age	20s	194 (19.4)	7,004,966 (18.8)
	30s	199 (19.9)	7,446,677 (20)
	40s	235 (23.5)	8,408,883 (22.6)
	50s	244 (24.4)	8,515,725 (22.9)
	60s	128 (12.8)	5,854,493 (15.7)

### Hand hygiene products used before and after the COVID-19 outbreak

The products normally used (bar soap, liquid hand soap, and hand sanitizer) for handwashing in Korea (1000 consumers were surveyed) pre- and post-COVID-19 were investigated and compared with Crème data [14], a study conducted on 448 American subjects. Before the pandemic, 41.4% of consumers used only bar soap, and 7.7% used only liquid hand soap. Among the consumers who used both bar soap and liquid hand soap, those who primarily used bar soap, liquid hand soap, or both bar soap and liquid hand soap were 30.4%, 15.8%, and 4.7%, respectively. After the outbreak of COVID-19, the proportion of consumers using only bar soap decreased to 17%, and the proportion of consumers using only liquid hand soap increased to 13.6%. The Crème data show that before the pandemic, American consumers mainly used liquid hand soap (69%, compared to 21% who mainly used bar soap); in Korea, 71.8% of consumers mainly used bar soap before the pandemic, and 51.4% continued to use it after the outbreak. Therefore, both percentages of bar soap users are higher compared with consumers that primarily use liquid hand soap (Table 2). The cross-tabulation test showed significant differences (*p*-value = 1.54E−230) in the products used before and after the outbreak of COVID-19 (Table 3).

**Table 2:** Comparison of the products usually used to wash hands

Usual used to wash hand	Crème	This study (pre)	This study (post)
	*N* (%)	*N* (%)	*N* (%)
Only bar soap	55 (12)	414 (41.4)	170 (17)
Primarily bar soap	41 (9)	304 (30.4)	344 (34.4)
Only liquid hand soap	232 (52)	77 (7.7)	136 (13.6)
Primarily liquid hand soap	77 (17)	158 (15.8)	277 (27.7)
Bar soap and liquid hand soap equally	14 (3)	47 (4.7)	73 (7.3)
None of the above	29 (6)	–	–
Total	448 (100)	1000 (100)	1000 (100)

**Table 3:** Usual product used to wash hands before and after the outbreak of COVID-19

Post	Only bar soap	Primarily bar soap	Only liquid hand soap	Primarily liquid hand soap	Bar soap and liquid hand soap equally	Total
Pre						
Only bar soap	157	161	24	56	16	414
Primarily bar soap	9	174	14	91	16	304
Only liquid hand soap	2	3	65	7	0	77
Primarily liquid hand soap	1	4	32	116	5	158
Bar soap and liquid hand soap equally	1	2	1	7	36	47
Total	170	344	136	277	73	1000

The *p*-value pre- and post-COVID-19 is less than 0.001.

### Use and carrying of hand sanitizer before and after the outbreak

We performed McNemar’s test to investigate changes in the use and carrying of hand sanitizer before and after the COVID-19 outbreak. Both the use and carrying of hand sanitizer had a *p*-value < 2.2E−16, and a significant change was detected between pre- and post-COVID-19 use. The number of consumers using hand sanitizer increased from 14.6% pre-COVID-19 to 89.8% post-COVID-19 (Table 4), and the number of consumers carrying hand sanitizer increased from 4.1% pre-COVID-19 to 39.3% post-COVID-19 (Table 5).

**Table 4:** Consumer use of hand sanitizer pre- or post-COVID-19

Post	No use	Use	Total
Pre			
Not used	89	765	854
Used	13	133	146
Total	102	898	1000

The *p*-value pre- and post-COVID-19 is less than 0.001

**Table 5:** Consumer carrying of hand sanitizer pre- or post-COVID-19

Post	Yes	No	Total
Pre			
Yes	33	8	41
No	360	599	959
Total	393	607	1000

The *p*-value pre- and post-COVID-19 is less than 0.001

### Co-use combinations of bar soap, liquid hand soap and hand sanitizer

Changes in co-use combinations of bar soap, liquid hand soap, and hand sanitizer were analyzed. The six co-use combinations of the three products are shown in Table 6. Before COVID-19, the rates of those who used only bar soap and only liquid hand soap are 37.6% and 31.7%, respectively, followed by those who used both bar soap and liquid hand soap (16.1%). After the COVID-19 outbreak, the rate of respondents who use bar soap, liquid hand soap, and hand sanitizer is the highest at 65.5% and the rates of those who use only bar soap and only liquid hand soap are 4.7% and 1.6%, respectively. Those who now use a combination of bar soap and liquid hand soap account for 3.9%. The proportion of those who used a combination of bar soap and hand sanitizer before COVID-19 is the lowest at 3.8%, but this increased to 12.3% after the outbreak. The changes in co-use combination of the three hand hygiene products by age groups and by occupational groups are shown in Additional file 2: Tables S2 and S3.

**Table 6:** Ratio of co-use of bar soap, liquid hand soap and hand sanitizer

Pre		Post	
Combination	Prop (%)	Combination	Prop (%)
Bar soap	37.6	Bar soap, liquid hand soap, hand sanitizer	65.5
Liquid hand soap	31.7	Bar soap, hand sanitizer	12.3
Bar soap, liquid hand soap	16.1	Liquid hand soap, hand sanitizer	12
Liquid hand soap, hand sanitizer	6.4	Bar soap	4.7
Bar soap, liquid hand soap, hand sanitizer	4.4	Bar soap, liquid hand soap	3.9
Bar soap, hand sanitizer	3.8	Liquid hand soap	1.6

### Comparison of bar soap, liquid hand soap and hand sanitizer usage patterns pre- and post-COVID-19

**Fig. 1:**
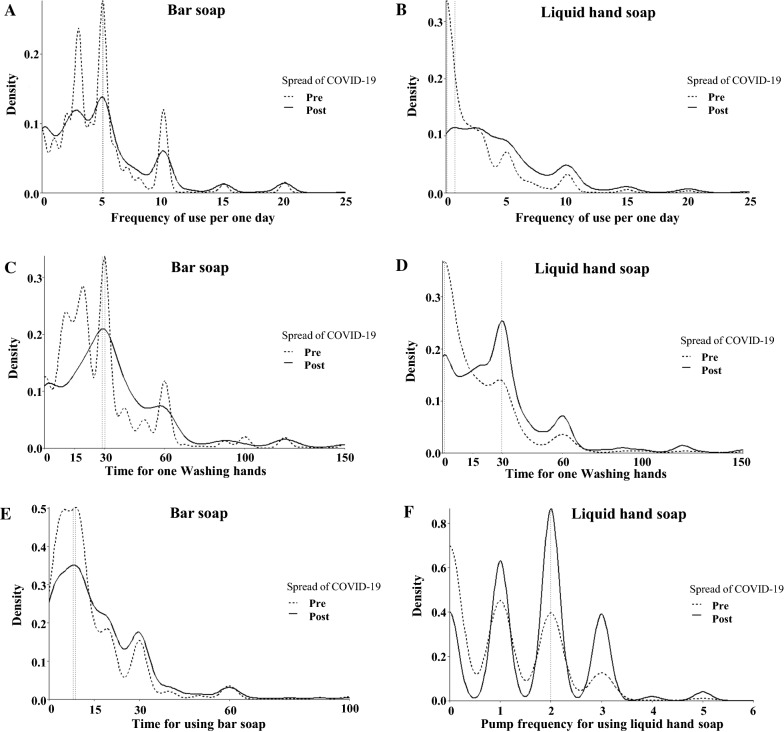
Comparison of bar soap and liquid hand soap usage patterns before and after the outbreak of COVID-19 in the form of density plots. **A** Frequency of bar soap use per one day, **B** frequency of liquid hand soap use per one day, **C** duration of handwashing while using bar soap, **D** duration of handwashing while using liquid hand soap, **E** time for rubbing bar soap, **F** number of pumps of liquid hand soap used

We analyzed the frequency, handwashing duration, and amount of product used through a density plot that shows consumer usage patterns. Peaks were analyzed for users and non-users of each product. The frequency of bar soap use revealed multiple peaks pre- and post-COVID-19 at three, five, and ten times per day; the highest peak was at five times per day (Fig. 1A). Approximately 92% of consumers who did not use liquid hand soap pre-COVID-19 slightly increased their use to approximately one time per day after the outbreak (Fig. 1B). The duration of handwashing with bar soap peaked at 30 s pre-COVID-19 with a marginal change to 29 s post-COVID-19 outbreak (Fig. 1C). The duration of handwashing with liquid hand soap pre- and post-COVID-19 outbreak peaked at 1 s and 30 s, respectively (Fig. 1D). This increase indicates that the public is abiding by the recommendations of the regulatory authorities. The duration of rubbing bar soap on the hand was approximately 9 s pre-COVID-19 and 8 s post-COVID-19 (Fig. 1E). The peak of the number of liquid hand soap pumps was zero pre-COVID-19 and approximately two pumps post-COVID-19 (Fig. 1F).

**Fig. 2:**
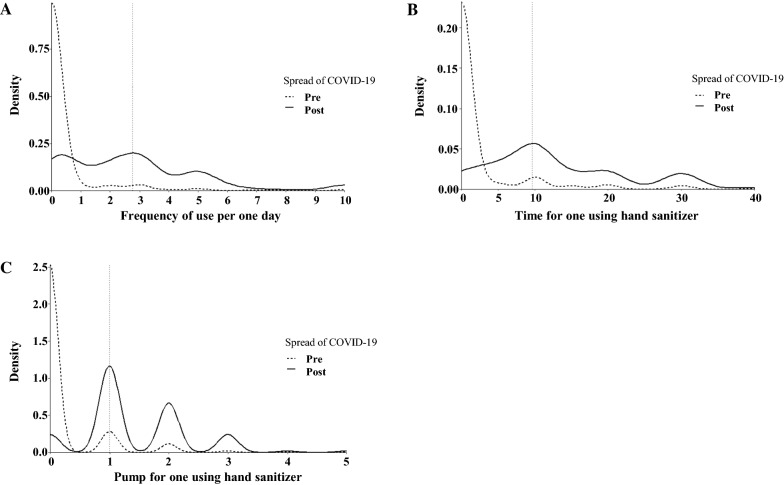
Comparison of hand sanitizer usage patterns before and after the COVID-19 outbreak in the form of density plots. **A** Frequency of use per day, **B** duration of hand sanitizer use, **C** number of pumps of hand sanitizer used

Most consumers (95.9%) did not use hand sanitizer before the COVID-19 outbreak. The peak in frequency of use was once per day, the duration of use was 0 s, and number of pumps was zero. The second-highest peak for the duration of use and number of pumps used for hand sanitizer was 10 s and one pump, respectively. After the outbreak, the peak was three times per day, 10 s duration, and one pump (Fig. 2).

### Patterns of handwashing and product usage

We analyzed how many times during the day the respondents washed their hands and how long it takes for them to wash only their hands. The analysis excludes those who responded “no use”. The average frequency of handwashing per day increased from 6.96 times to 9.98 times after the COVID-19 outbreak. Similarly, the average time taken to wash one’s hands increased from 20.16 to 31.58 s (Table 7). According to the Korean exposure factor handbook data [15], handwashing duration was 78 s during the summer and winter, which is higher than the duration found in this study. We asked consumers to measure the actual time they spend washing their hands. In addition, after COVID-19 outbreak, the frequency and duration of handwashing had a significant increase in all age groups (20s, 30s, 40s, 50s and 60s) (Additional file 2: Table S4) and in some occupational groups (professionals; business/management/office; sales/service; production/technical; housewife; student; unemployment/leave) out of 13 job categories (Additional file 2: Table S5). The statistics show that the frequency, duration, and amount of product used reported for bar soap, liquid hand soap, and hand sanitizer after the COVID-19 outbreak are significantly different from those reported before the outbreak (Additional file 2: Table S4).

**Table 7:** Comparison of handwashing patterns

Study	Handwashing	Period	P5	P25	P50	P75	P95	Mean	SD
This study	Frequency	Pre	2.00	4.00	6.00	9.00	15.00	6.96	4.52
	Frequency	Post	3.00	5.00	8.00	12.00	22.00	9.98	7.27
	Time	Pre	3.00	8.00	13.33	23.33	60.00	20.16	24.15
	Time	Post	5.00	11.67	21.67	31.04	95.25	31.58	38.71
Korean Exposure Factors Handbook (2019)	Frequency	Summer	–	3.00	5.00	7.00	10.00	5.60	2.80
	Frequency	Winter	–	3.00	4.00	5.00	10.00	4.60	2.40
	Time	Summer	–	54.00	60.00	120.00	180.00	78.00	48.00
	Time	Winter	–	30.00	60.00	120.00	180.00	78.00	48.00
USEPA (2011)	Frequency	–	–	–	–	–	–	0.14–2.57	–
Norden (2012)	Frequency	–	–	–	–	–	–	3–6	–

It was found that the highest proportion of Korean consumers (20% and 23.3%) washed their hands five times a day pre-COVID-19, and they wash 11–20 times a day post-COVID-19. According to Crème data [14], 21% of respondents wash their hands 5 times a day, and an almost equal proportion (19%) wash their hands 10 times a day (Table 8).

**Table 8:** Comparison of the typical frequency of handwashing

Frequency of washing hands	Crème	This study (Pre)	This study (Post)
	*N* (%)	*N* (%)	*N* (%)
1 time per day	5 (1.1)	11 (1.1)	4 (0.4)
2 times per day	14 (3.1)	42 (4.2)	12 (1.2)
3 times per day	35 (7.8)	121 (12.1)	50 (5.0)
4 times per day	48 (10.7)	117 (11.7)	50 (5.0)
5 times per day	95 (21.2)	200 (20.0)	130 (13.0)
6 times per day	52 (11.6)	106 (10.6)	95 (9.5)
7 times per day	16 (3.6)	81 (8.1)	88 (8.8)
8 times per day	26 (5.8)	68 (6.8)	108 (10.8)
9 times per day	5 (1.1)	18 (1.8)	38 (3.8)
10 times per day	85 (19.0)	105 (10.5)	134 (13.4)
11–20 times per day	52 (11.6)	118 (11.8)	233 (23.3)
21–30 times per day	10 (2.2)	11 (1.1)	45 (4.5)
31–40 times per day	1 (0.2)	2 (0.2)	7 (0.7)
41–50 times per day	1 (0.2)	0 (0.0)	3 (0.3)
50 + times per day	1 (0.2)	0 (0.0)	3 (0.3)
Don't wash hands	2 (0.44)	–	–
Total	448	1,000	1,000

### Consumer perception of the adverse health effects and the preventive effects of the three products

The latter part of the questionnaire asked the respondents about their use of hand-washing products before and after the COVID-19 outbreak (Q8, 10, 12 in the questionnaire). The mean, standard deviation, and *p*-values of the *t*-test are presented in Table 9. Concerns about the adverse health effects for bar soap increased significantly from 2.51 before COVID-19 to 2.77 after. By contrast, the concern for hand sanitizers changed from 3.24 to 3.17, while the concern for liquid hand soap showed no change. Respondents were asked to rate how effective these products were in preventing the spread of COVID-19 on a 7-point scale (Table 10). Analysis of variance was performed on the three products, and the average values of perception differed (*F*-value = 1.0E−4). As a result of the *t*-test for the two products, bar and liquid hand soap differed in average value with a *p*-value of 1.15E−4. Bar soap and hand sanitizer also showed a difference in average value with a *p*-value of 4.693E−4. However, the perceived preventive effect of liquid hand soap and hand sanitizer did not differ (*p*-value = 0.901). The perceived preventive effect against COVID-19 was higher for liquid hand soap and hand sanitizer than it was for bar soap.

**Table 9:** Comparison of hazards about the adverse effects of products use before and after the COVID-19 outbreak

Product	COVID-19	Mean	SD	*p*-value
Bar soap***	Pre	2.51	1.361	0.000
	Post	2.77	1.617	
Liquid hand soap	Pre	2.88	1.456	0.133
	Post	2.84	1.549	
Hand sanitizer*	Pre	3.24	1.486	0.020
	Post	3.17	1.591	

*p-value < 0.05, **p-value < 0.01 and ***p-value < 0.001

**Table 10:** Perception on the preventive effect against COVID-19 of products

Product	Mean	SD	*F*-value
Bar soap	5.62	1.062	1.E−04
Liquid hand soap	5.78	0.883	
Hand sanitizer	5.77	0.925	

The *F*-value is 0.0001. The *p*-value of bar soap and liquid hand soap, or hand sanitizer is less than 0.001. The *p*-value of liquid hand soap and hand sanitizer is 0.9016

## Discussion

In the wake of the COVID-19 outbreak, frequent handwashing and proper hand hygiene are strongly recommended by international health agencies and Korean regulatory agencies, such as the Korean Center for Disease Control and the Ministry of Food and Drug Safety, as the easiest, most economic, and most effective ways to prevent the spread of the virus. The Centers for Disease Control and Prevention (CDC) recommends washing hands with soap and water for at least 20 s or using a hand sanitizer with at least 60% alcohol to prevent the spread of germs during the COVID-19 pandemic [16]. The hand hygiene behavior described in this study indicates that the frequency of handwashing has increased from 6.96 times to 9.98 times per day during the COVID pandemic, and the average handwashing duration also increased from 20.16 to 31.59 s. Access to hand-washing facilities was analyzed worldwide, and the analysis suggested the need for either improving access to handwashing facilities or providing alternative methods for handwashing in low-income countries [17]. Only 0.9% (95% uncertainty interval [UI]: 0.5, 1.5) of the population lacked access to handwashing facilities, with access to soap and water in 2019 compatible with that found in North America, where only 0.4% (95% UI: 0.3, 9.5) of the population lacked access [17]. However, a previous report showed that the estimated actual prevalence of handwashing behavior in Korea was only 17% (95% UI: 9, 33) compared to 49% in the United States [18]. This is strikingly low considering the access to handwashing facilities in Korea. Our survey only measures the behavior and attitudes of those who responded to washing hands, so the prevalence of handwashing was not evaluated.

A total of 1123 participants were dropped from the survey, either because they did not complete it or responded “no wash”. The results do not distinguish between those who responded “no wash” and those who did not complete the survey. Therefore, at most, 52% (1123/2123) of the survey respondents do not wash their hands. A 2014 study by Freeman et al. [18] may have underestimated handwashing prevalence, but if their finding of 17% is accurate, it indicates that handwashing behavior has improved to 48% in 2020. Interestingly, the average daily frequency at which Koreans wash their hands indicated by our data and found in the Korean exposure factor handbook (2019) [15] was relatively high compared to data from USEPA [19] and Norden [20] (7). These results should be considered when establishing public guidelines and policies. Guidelines that emphasize the importance of washing one’s hands and increasing the prevalence of handwashing behavior is needed more than guidelines that state how to wash and for how long. A survey from Europe showed that more than 95% of individuals wash their hands with soap and water more frequently after the COVID-19 outbreak than they did before [21]. Therefore, the COVID-19 pandemic presents a good opportunity to develop enforcement measures to increase handwashing and improve public hygiene.

The number of Korean consumers who use liquid hand soap has increased (from 23.5% before the outbreak to 41.3% after). Although, many consumers still use bar soap (51.4%), the number of consumers using only bar soap decreased significantly from 41.4% before the pandemic to 17% after (2). As shown in Table 10, consumer perception of bar soap’s preventive effect was lower than that of liquid hand soap. Korean consumers likely prefer to use liquid hand soap instead of bar soap, because liquid soap is perceived to be more effective and less contaminated than bar soap, which is fully exposed at handwashing facilities [22]. Despite the preference for liquid hand soap, the higher number of consumers who primarily use bar soap is due to the lack of liquid hand soap at handwashing facilities. It is interesting to point out that one’s perception of the soap available and the effectiveness of hand hygiene agents greatly influences personal handwashing behavior. Therefore, it is necessary to increase the supply of liquid hand soap at handwashing facilities, to provide guidance on the proper use of liquid hand soap and information related to safe liquid hand soap product because consumer opinion of liquid hand soap is higher than that of bar soap (9).

The use of hand sanitizers containing biocidal alcohol (i.e., ethanol and various biocidal agents) among Korean consumers increased from 14.6 to 89.8%. In addition, the number of consumers carrying hand sanitizer increased from 4.1 to 39.3%. This change is due to consumers’ perception that hand sanitizers are more effective at killing the virus that causes COVID-19 than handwashing with soap. By contrast, 9 shows that concern over the hazardous effects of hand sanitizer has decreased since the COVID-19 outbreak. However, WHO recommends that soap and alcohol-based hand sanitizers should not be used concomitantly [23], and questions related to the potential risk of hand sanitizer products are emerging with their increased use. The main ingredient in hand sanitizer is either ethanol (80%) or isopropyl alcohol (75%); limited amounts of hydrogen peroxide, benzalkonium chloride, or sodium hypochlorite may also be present. A high concentration of sodium hypochlorite can cause chemical burns [23], inhaling a high concentration of alcohol vapor can cause respiratory irritation and affect the central nervous system [24], and continuous contact with quaternary ammonium compounds can irritate the skin and cause acute toxicity such as difficulty breathing [25]. In particular, methanol can have toxic effects when ingested or absorbed through the skin. With the ease of do-it-yourself hand sanitizers recipes and possible loose legislation, countermeasures against the potential risks associated with improper use of hand sanitizers are needed. The decrease in consumer concern about the hazardous effects associated with the use of hand sanitizers is illustrated in 9.

In Korea, humidifier disinfectants containing biocidal ingredients caused 6246 reported cases of and 1375 deaths by pulmonary fibrosis as of February 28, 2018 [26-28]. These incidents have raised a huge wave of concerns regarding exposure to and damage caused by chemicals. Strict guidelines and legislation for hand sanitizers are required to prevent related accidents during this pandemic. Tools are available to provide hazard information on consumer products in other countries, such as the German ‘ToxFox [29]’, Danish ‘Kemiluppen [30]’, and the American ‘GoodGuide [31]’. Our group is also developing a platform to provide risk information about the aggregate use of personal and household products. These tools can be used to provide continuous updates and information regarding personal hygiene care products and prevent their inappropriate use. A recent study reported that exposure to infographics and infographics plus videos through an evidence-based public health campaign is associated with better personal hygiene, which includes handwashing [32]. Access to personal hygiene data through certain media, such as smartphone apps, that show the percentile status of one’s frequency and, duration of handwashing, and their co-use of hand sanitizer, will increase personal hygienic behavior.

## Conclusions

In the present study, we examine Korean behavior patterns related to bar soap, liquid hand soap, and hand sanitizer before and after the COVID-19 outbreak, as well as consumer concerns regarding the safety of the three products studied and consumer perception of the products’ effectiveness in preventing the spread of COVID-19. The data collected not only provide insight into consumer behavior patterns, such as the frequency of use, the duration of use, and the amount of product used, but they also show how these patterns have changed since the COVID-19 outbreak. As consumer use of hand sanitizers has increased, public health guidelines must address the potential risks associated with them. Our data also show that the consumers are abiding by the recommendations of the regulatory authorities for handwashing. As handwashing has become important in preventing COVID-19 infections, our analysis of changes in handwashing behavior that occurred after the COVID-19 outbreak will support the development of better handwashing guidelines and a public health campaign.

## Supplementary Information

**Additional file 1:** Questions for selecting respondents

**Additional file 2: Table S1.** Comparison of bar soap, liquid hand soap and hand sanitizer usage patterns of all respondents pre- and post-COVID-19. **Table S2.** Ratio of co-use of bar soap, liquid hand soap and hand sanitizer before and after the outbreak of COVID-19 by age. **Table S3.** Ratio of co-use of bar soap, liquid hand soap and hand sanitizer before and after the outbreak of COVID-19 by occupation. **Table S4.** Comparison of average handwashing frequency and average handwashing duration pre- and post-COVID-19 by age. **Table S5.** Comparison of average handwashing frequency and average handwashing duration pre- and post-COVID-19 by occupation. **Table S6**. Comparison of bar soap, liquid hand soap and hand sanitizer usage patterns of consumers using products pre- and post-COVID-19.

## Data Availability

Not applicable.
