# Supplementary material for: Changes in handwashing and hygiene product usage patterns in Korea before and after the outbreak of COVID-19
Source: Environ Sci Eur. 2021 Jul 3;33(1):79. doi: 10.1186/s12302-021-00517-8 (PMC8254429; doi:10.1186/s12302-021-00517-8)
Supplement: Supplementary file 1 — Additional file 1: Questions for selecting respondents [file 12302_2021_517_MOESM1_ESM.docx]

**Social Problem Solving Project**

**Developing a Platform for Safety information on**

**Consumer Chemical Products**

We are performing a research on 'Development of a platform to provide safety information on consumer chemical products' supported by the Ministry of Science and ICT and the National Research Foundation as one of the 'Technology development projects to solve social problems'. For the purpose, we are administering this survey to help the research to determine the safety of consumer chemical products when used continuously. The results will be used as precious data to develop a more accurate application. As such, we appreciate your kind assistance. Thank you.

| Questions for selecting respondents |
| --- |

SQ1. Are you male or female? ① Male ② Female

SQ2. How old are you? ( )

SQ3. Where do you currently live?

1 Seoul 2 Busan 3 Daegu 4 Incheon

5 Gwangju 6 Daejeon 7 Ulsan 8 Sejong

9 Gyeonggi-do 10 Gangwon-do 11 Chungcheongbuk-do

12 Chungcheongnam-do 13 Jeonrabuk-do 14 Jeonranam-do

15 Gyeongsangbuk-do 16 Gyeongsangnam-do 17 Jeju-do

SQ4. How did you wash your hands **before the COVID-19 spread**?

| Bar soap | Liquid hand soap |
| --- | --- |
| 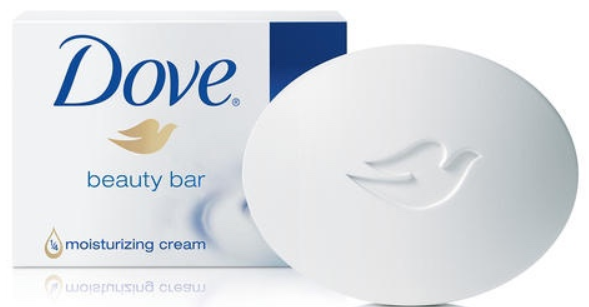 | 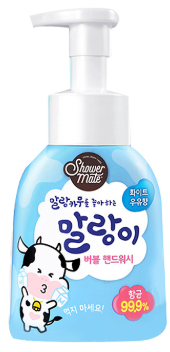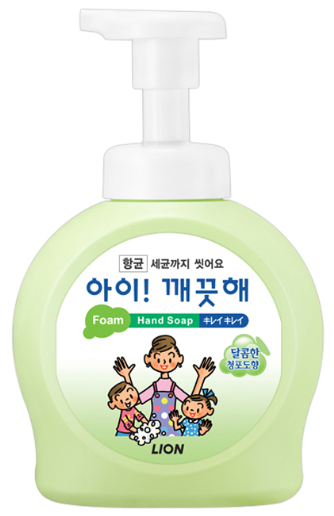 |

① Only bar soap

② Only liquid hand soap

③ Both bar soap and liquid hand soap, but primarily bar soap

④ Both bar soap and liquid hand soap, but primarily liquid hand soap

⑤ Equally bar soap and liquid hand soap

⑥ Neither was used

SQ5. How did you wash your hands **after the COVID-19 spread**?

① Only bar soap

② Only liquid hand soap

③ Both bar soap and liquid hand soap, but primarily bar soap

④ Both bar soap and liquid hand soap, but primarily liquid hand soap

⑤ Equally bar soap and liquid hand soap

⑥ Neither is used

SQ6. Do you use hand sanitizer?

| 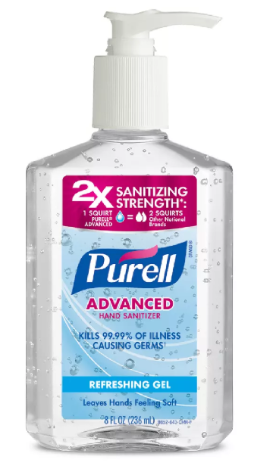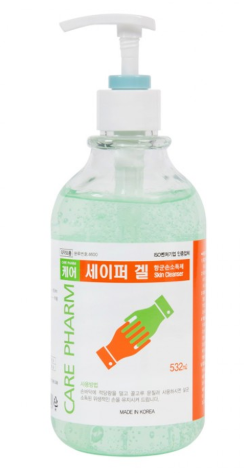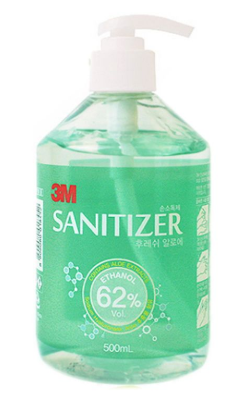 |
| --- |

① Not used both before and after COVID-19

② Used only before COVID-19 and not after

③ Used only after COVID-19 and not before

④ Used both before and after COVID-19

SQ7-1. Did you carry hand sanitizers when you went out **before the COVID-19 spread**?

① Carried ② Not carried

SQ7-2. Do you carry hand sanitizers when you go out **after the COVID-19 spread**?

① Carry ② Not carry

| **A.** |
| --- |

Q1-1. How many times did you wash your hands with bar soap and liquid hand soap a day **before the COVID-19 spread**? (※ Respond based on one month prior to the spread of COVID-19)

| 1) Bar soap | ( ) times a day |
| --- | --- |
| 2) Liquid hand soap | ( ) times a day |

Q1-2. How many times do you wash your hands with bar soap and liquid hand soap a day **after the COVID-19 spread**? (※ Respond based on one month following the spread of COVID-19)

| 1) Bar soap | ( ) times a day |
| --- | --- |
| 2) Liquid hand soap | ( ) times a day |

Q2-1. How long did you wash your hands using bar soap at a time **before the COVID-19 spread**? (※ Respond based on one month prior to the spread of COVID-19)

| ※ Cleaning time is the entire time you lather and rinse.  ※ Usage time is time you lather |
| --- |

| 1) Cleaning time: ( ) seconds |
| --- |
| 2) Usage time: ( ) seconds |

Q2-2. How long do you wash your hands using bar soap at a time **after the COVID-19 spread**? (※ Respond based on one month following the spread of COVID-19)

| ※ Cleaning time is the entire time you lather and rinse.  ※ Usage time is time you lather  **※ Please measure the cleaning time and usage time and respond** |
| --- |

| 1) Cleaning time: ( ) seconds |
| --- |
| 2) Usage time: ( ) seconds |

Q3-1. How much did you pump for a single use of liquid hand soap **before the COVID-19 spread**? (※ Respond based on one month prior to the spread of COVID-19)

( ) pumps in once

Q3-2. How much do you pump for a single use of liquid hand soap **after the COVID-19 spread**? (※ Respond based on one month following the spread of COVID-19)

( ) pumps in once

Q4-1. How long did you wash your hands using liquid hand soap at a time **before the COVID-19 spread**? (※Respond based on one month prior to the spread of COVID-19)

| ※ Cleaning time is the entire time you lather and rinse. |
| --- |

( ) seconds

Q4-2. . How long do you wash your hands using liquid hand soap at a time **after the COVID-19 spread**? (※Respond based on one month following the spread of COVID-19)

| ※ Cleaning time is the entire time you lather and rinse.  **※ Please measure the cleaning time and usage time and respond** |
| --- |

( ) seconds

Q5-1. How many times did you use hand sanitizer a day **before the COVID-19 spread**? (※ Respond based on one month prior to the spread of COVID-19)

| 0.5 times a day if used once every 2 days |
| --- |
| 0.3 times a day if used once every 3-4 days |
| 0.2 times a day if used once 5 to 6 days |
| 0.1 times a day if used once a week |

( ) times a day

Q5-2. How many times do you use hand sanitizer a day **after the COVID-19 spread**? (※ Respond based on one month following the spread of COVID-19)

| 0.5 times a day if used once every 2 days |
| --- |
| 0.3 times a day if used once every 3-4 days |
| 0.2 times a day if used once 5 to 6 days |
| 0.1 times a day if used once a week |

( ) times a day

Q6-1. How much did you pump for a single use of hand sanitizer **before the COVID-19 spread**? (※ Respond based on one month prior to the spread of COVID-19)

( ) pumps in once

Q6-2. How much do you pump for a single use of hand sanitizer **after the COVID-19 spread**? (※ Respond based on one month following the spread of COVID-19)

( ) pumps in once

Q7-1. How long did you rub your hands when using hand sanitizer at a time **before the COVID-19 spread**? (※Respond based on one month prior to the spread of COVID-19)

( ) seconds

Q7-2. How long do you rub your hands when using hand sanitizer at a time **after the COVID-19 spread**? (※Respond based on one month following the spread of COVID-19)

| **※ Please measure the usage time and respond** |
| --- |

( ) seconds

Q8-1. How dangerous/harmful did you think bar soap was to you **before the COVID-19 spread**? (※ Respond based on one month prior to the spread of COVID-19)

| Totally harmless | Harmless | Slightly harmless | Neutral | Slightly harmful | Harmful | Totally harmful |
| --- | --- | --- | --- | --- | --- | --- |
| ① | ② | ③ | ④ | ⑤ | ⑥ | ⑦ |

Q8-2. How dangerous/harmful do you think bar soap is to you **after the COVID-19 spread**? (※ Respond based on one month following the spread of COVID-19)

| Totally harmless | Harmless | Slightly harmless | Neutral | Slightly harmful | Harmful | Totally harmful |
| --- | --- | --- | --- | --- | --- | --- |
| ① | ② | ③ | ④ | ⑤ | ⑥ | ⑦ |

Q9. How effective do you think bar soap use will be to prevent COVID-19?

| Totally Ineffective | Ineffective | Slightly ineffective | Neutral | Slightly effective | Effective | Totally effective |
| --- | --- | --- | --- | --- | --- | --- |
| ① | ② | ③ | ④ | ⑤ | ⑥ | ⑦ |

Q10-1. How dangerous/harmful did you think liquid hand soap was to you **before the COVID-19 spread**? (※Respond based on one month prior to the spread of COVID-19)

| Totally harmless | Harmless | Slightly harmless | Neutral | Slightly harmful | Harmful | Totally harmful |
| --- | --- | --- | --- | --- | --- | --- |
| ① | ② | ③ | ④ | ⑤ | ⑥ | ⑦ |

Q10-2. How dangerous/harmful do you think liquid hand soap is to you **after the COVID-19 spread**? (※ Respond based on one month following the spread of COVID-19)

| Totally harmless | Harmless | Slightly harmless | Neutral | Slightly harmful | Harmful | Totally harmful |
| --- | --- | --- | --- | --- | --- | --- |
| ① | ② | ③ | ④ | ⑤ | ⑥ | ⑦ |

Q11. How effective do you think liquid hand soap use will be to prevent COVID-19?

| Totally Ineffective | Ineffective | Slightly ineffective | Neutral | Slightly effective | Effective | Totally effective |
| --- | --- | --- | --- | --- | --- | --- |
| ① | ② | ③ | ④ | ⑤ | ⑥ | ⑦ |

Q12-1. How dangerous/harmful did you think hand sanitizer was to you **before the COVID-19 spread**? (※ Respond based on one month prior to the spread of COVID-19)

| Totally harmless | Harmless | Slightly harmless | Neutral | Slightly harmful | Harmful | Totally harmful |
| --- | --- | --- | --- | --- | --- | --- |
| ① | ② | ③ | ④ | ⑤ | ⑥ | ⑦ |

Q12-2. How dangerous/harmful do you think hand sanitizer is to you **after the COVID-19 spread**? (※ Respond based on one month following the spread of COVID-19)

| Totally harmless | Harmless | Slightly harmless | Neutral | Slightly harmful | Harmful | Totally harmful |
| --- | --- | --- | --- | --- | --- | --- |
| ① | ② | ③ | ④ | ⑤ | ⑥ | ⑦ |

Q13. How effective do you think hand sanitizer use will be to prevent COVID-19?

| Totally Ineffective | Ineffective | Slightly ineffective | Neutral | Slightly effective | Effective | Totally effective |
| --- | --- | --- | --- | --- | --- | --- |
| ① | ② | ③ | ④ | ⑤ | ⑥ | ⑦ |

| B. Demographics of respondents |
| --- |

BQ1. Are you married? ① Yes ② No

BQ2. How many family members do you have? (Including yourself) ( )

BQ3. (For those who answered ① at DQ 1.) Do you have child?

① Yes (→ BQ3-1) ② No (→ BQ4)

BQ3-1. How many children do you have? ( )

BQ3-1. How many minor children do you have? ( )

BQ4. What is the average monthly personal income? ( )

BQ4-1. What is the average monthly household income? ( )

| ※ The average monthly household income is the sum of  all household members' earned income, interest, pension, and rental income. |
| --- |

BQ5. What is your current occupation?

① Professional/administrative position ② White-collar (management, office job)

③ Self-employment ④ Sales/service ⑤ Blue-collar (production)

⑥ Farming (agriculture/livestock/fishery) ⑦ Housemaker ⑧ Student

⑨ Other ( )

BQ6. How tall are you? ( ) cm

BQ7. How much do your weight? ( ) kg

BQ8. How large is your house?

| ※ 1 pyeong = 3.3 m^2^ |
| --- |

① ( ) pyeong ② ( ) m^2^

Thanks for your time.
