# Supplementary material for: Changes in handwashing and hygiene product usage patterns in Korea before and after the outbreak of COVID-19
Source: Environ Sci Eur. 2021 Jul 3;33(1):79. doi: 10.1186/s12302-021-00517-8 (PMC8254429; doi:10.1186/s12302-021-00517-8)
Supplement: Supplementary file 2 — Additional file 2: Table S1. Comparison of bar soap, liquid hand soap and hand sanitizer usage patterns of all respondents pre- and post-COVID-19. Table S2. Ratio of co-use of bar soap, liquid hand soap and hand sanitizer before and after the outbreak of COVID-19 by age. Table S3. Ratio of co-use of bar soap, liquid hand soap and hand sanitizer before and after the outbreak of COVID-19 by occupation. Table S4. Comparison of average handwashing frequency and average handwashing duration pre- and post-COVID-19 by age. Table S5. Comparison of average handwashing frequency and average handwashing duration pre- and post-COVID-19 by occupation. Table S6. Comparison of bar soap, liquid hand soap and hand sanitizer usage patterns of consumers using products pre- and post-COVID-19. [file 12302_2021_517_MOESM2_ESM.docx]

**Table S1** Comparison of bar soap, liquid hand soap and hand sanitizer usage patterns of all respondents pre- and post-COVID-19.

| **Usage pattern** | **Product** | **COVID-19** | **Mean** | **SD** | **Peak** | **p-value** |
| --- | --- | --- | --- | --- | --- | --- |
| **Frequency** | **Bar soap*** | **pre** | **4.689** | **3.707** | **5.004** | **0.0264** |
|  |  | **post** | **5.129** | **5.046** | **4.956** |  |
|  | **Liquid hand soap***** | **pre** | **2.275** | **3.14** | **0.059** | **<2.2E-16** |
|  |  | **post** | **4.851** | **5.528** | **0.739** |  |
|  | **Hand sanitizer***** | **pre** | **0.346** | **1.582** | **0** | **<2.2E-16** |
|  |  | **post** | **2.919** | **3.107** | **2.745** |  |
| **Time** | **Bar soap***** | **pre** | **32.164** | **36.241** | **30.285** | **5.27E-06** |
|  |  | **post** | **41.199** | **50.987** | **28.684** |  |
|  | **Liquid hand soap***** | **pre** | **17.258** | **28.897** | **0.979** | **<2.2E-16** |
|  |  | **post** | **32.802** | **43.375** | **29.495** |  |
|  | **Hand sanitizer***** | **pre** | **2.064** | **6.397** | **0** | **<2.2E-16** |
|  |  | **post** | **14.514** | **14.436** | **9.597** |  |
| **Amount** | **Bar soap***** | **pre** | **14.589** | **18.566** | **8.833** | **1.18E-05** |
|  |  | **post** | **19.226** | **27.73** | **8.052** |  |
|  | **Liquid hand soap***** | **pre** | **1.004** | **1.045** | **0** | **<2.2E-16** |
|  |  | **post** | **1.643** | **1.134** | **1.994** |  |
|  | **Hand sanitizer***** | **pre** | **0.213** | **0.593** | **0** | **<2.2E-16** |
|  |  | **post** | **1.462** | **0.958** | **0.993** |  |

*p-value<0.05, ** p-value <0.01 and *** p-value <0.001

**Table S2** Ratio of co-use of bar soap, liquid hand soap and hand sanitizer before and after the outbreak of COVID-19 by age.

| **20s** | | | |
| --- | --- | --- | --- |
| **pre** | | **post** | |
| **combination** | **%** | **combination** | **%** |
| **Bar soap** | **58.2** | **Bar soap** | **11.9** |
| **Liquid hand soap** | **4.6** | **Liquid hand soap** | **4.6** |
| **Bar soap, Liquid hand soap** | **21.6** | **Bar soap, Liquid hand soap** | **6.2** |
| **Bar soap, Hand sanitizer** | **8.8** | **Bar soap, Hand sanitizer** | **30.4** |
| **Liquid hand soap, Hand sanitizer** | **1.0** | **Liquid hand soap, Hand sanitizer** | **12.4** |
| **Bar soap, Liquid hand soap, Hand sanitizer** | **5.7** | **Bar soap, Liquid hand soap, Hand sanitizer** | **34.5** |
| **30s** | | | |
| **pre** | | **post** | |
| **combination** | **%** | **combination** | **%** |
| **Bar soap** | **51.8** | **Bar soap** | **9.5** |
| **Liquid hand soap** | **9.5** | **Liquid hand soap** | **4.5** |
| **Bar soap, Liquid hand soap** | **22.1** | **Bar soap, Liquid hand soap** | **11.6** |
| **Bar soap, Hand sanitizer** | **6.0** | **Bar soap, Hand sanitizer** | **27.6** |
| **Liquid hand soap, Hand sanitizer** | **3.0** | **Liquid hand soap, Hand sanitizer** | **15.6** |
| **Bar soap, Liquid hand soap, Hand sanitizer** | **7.5** | **Bar soap, Liquid hand soap, Hand sanitizer** | **31.2** |
| **40s** | | | |
| **pre** | | **post** | |
| **combination** | **%** | **combination** | **%** |
| **Bar soap** | **57.0** | **Bar soap** | **15.7** |
| **Liquid hand soap** | **9.4** | **Liquid hand soap** | **2.6** |
| **Bar soap, Liquid hand soap** | **19.1** | **Bar soap, Liquid hand soap** | **6.4** |
| **Bar soap, Hand sanitizer** | **10.2** | **Bar soap, Hand sanitizer** | **34.9** |
| **Liquid hand soap, Hand sanitizer** | **0.9** | **Liquid hand soap, Hand sanitizer** | **11.1** |
| **Bar soap, Liquid hand soap, Hand sanitizer** | **3.4** | **Bar soap, Liquid hand soap, Hand sanitizer** | **29.4** |
| **50s** | | | |
| **pre** | | **post** | |
| **combination** | **%** | **combination** | **%** |
| **Bar soap** | **75.4** | **Bar soap** | **13.5** |
| **Liquid hand soap** | **4.1** | **Liquid hand soap** | **0.8** |
| **Bar soap, Liquid hand soap** | **9.4** | **Bar soap, Liquid hand soap** | **4.9** |
| **Bar soap, Hand sanitizer** | **8.2** | **Bar soap, Hand sanitizer** | **50.0** |
| **Liquid hand soap, Hand sanitizer** | **0.0** | **Liquid hand soap, Hand sanitizer** | **7.8** |
| **Bar soap, Liquid hand soap, Hand sanitizer** | **2.9** | **Bar soap, Liquid hand soap, Hand sanitizer** | **23.0** |
| **60s** | | | |
| **pre** | | **post** | |
| **combination** | **%** | **combination** | **%** |
| **Bar soap** | **73.4** | **Bar soap** | **20.3** |
| **Liquid hand soap** | **3.9** | **Liquid hand soap** | **2.3** |
| **Bar soap, Liquid hand soap** | **5.5** | **Bar soap, Liquid hand soap** | **4.7** |
| **Bar soap, Hand sanitizer** | **13.3** | **Bar soap, Hand sanitizer** | **45.3** |
| **Liquid hand soap, Hand sanitizer** | **1.6** | **Liquid hand soap, Hand sanitizer** | **5.5** |
| **Bar soap, Liquid hand soap, Hand sanitizer** | **2.3** | **Bar soap, Liquid hand soap, Hand sanitizer** | **21.9** |

**Table S3** Ratio of co-use of bar soap, liquid hand soap and hand sanitizer before and after the outbreak of COVID-19 by occupation.

| **Professionals** | | | |
| --- | --- | --- | --- |
| **pre** | | **post** | |
| **combination** | **%** | **combination** | **%** |
| **Bar soap** | **48.2** | **Bar soap** | **9.1** |
| **Liquid hand soap** | **9.1** | **Liquid hand soap** | **4.5** |
| **Bar soap, Liquid hand soap** | **20.0** | **Bar soap, Liquid hand soap** | **10.9** |
| **Bar soap, Hand sanitizer** | **11.8** | **Bar soap, Hand sanitizer** | **31.8** |
| **Liquid hand soap, Hand sanitizer** | **1.8** | **Liquid hand soap, Hand sanitizer** | **10.9** |
| **Bar soap, Liquid hand soap, Hand sanitizer** | **9.1** | **Bar soap, Liquid hand soap, Hand sanitizer** | **32.7** |
| **Business/Management/Office** | | | |
| **pre** | | **post** | |
| **combination** | **%** | **combination** | **%** |
| **Bar soap** | **62.5** | **Bar soap** | **15.5** |
| **Liquid hand soap** | **7.6** | **Liquid hand soap** | **1.5** |
| **Bar soap, Liquid hand soap** | **16.4** | **Bar soap, Liquid hand soap** | **5.3** |
| **Bar soap, Hand sanitizer** | **9.7** | **Bar soap, Hand sanitizer** | **35.8** |
| **Liquid hand soap, Hand sanitizer** | **0.3** | **Liquid hand soap, Hand sanitizer** | **10.0** |
| **Bar soap, Liquid hand soap, Hand sanitizer** | **3.5** | **Bar soap, Liquid hand soap, Hand sanitizer** | **32.0** |
| **Self-employment** | | | |
| **pre** | | **post** | |
| **combination** | **%** | **combination** | **%** |
| **Bar soap** | **65.8** | **Bar soap** | **17.8** |
| **Liquid hand soap** | **4.1** | **Liquid hand soap** | **4.1** |
| **Bar soap, Liquid hand soap** | **16.4** | **Bar soap, Liquid hand soap** | **8.2** |
| **Bar soap, Hand sanitizer** | **9.6** | **Bar soap, Hand sanitizer** | **32.9** |
| **Liquid hand soap, Hand sanitizer** | **1.4** | **Liquid hand soap, Hand sanitizer** | **2.7** |
| **Bar soap, Liquid hand soap, Hand sanitizer** | **2.7** | **Bar soap, Liquid hand soap, Hand sanitizer** | **34.2** |
| **Sales/Service** | | | |
| **pre** | | **post** | |
| **combination** | **%** | **combination** | **%** |
| **Bar soap** | **62.9** | **Bar soap** | **14.4** |
| **Liquid hand soap** | **6.2** | **Liquid hand soap** | **2.1** |
| **Bar soap, Liquid hand soap** | **14.4** | **Bar soap, Liquid hand soap** | **8.2** |
| **Bar soap, Hand sanitizer** | **10.3** | **Bar soap, Hand sanitizer** | **40.2** |
| **Liquid hand soap, Hand sanitizer** | **1.0** | **Liquid hand soap, Hand sanitizer** | **15.5** |
| **Bar soap, Liquid hand soap, Hand sanitizer** | **5.2** | **Bar soap, Liquid hand soap, Hand sanitizer** | **19.6** |
| **Production/Technical** | | | |
| **pre** | | **post** | |
| **combination** | **%** | **combination** | **%** |
| **Bar soap** | **81.5** | **Bar soap** | **15.4** |
| **Liquid hand soap** | **0.0** | **Liquid hand soap** | **0.0** |
| **Bar soap, Liquid hand soap** | **7.7** | **Bar soap, Liquid hand soap** | **9.2** |
| **Bar soap, Hand sanitizer** | **4.6** | **Bar soap, Hand sanitizer** | **56.9** |
| **Liquid hand soap, Hand sanitizer** | **1.5** | **Liquid hand soap, Hand sanitizer** | **4.6** |
| **Bar soap, Liquid hand soap, Hand sanitizer** | **4.6** | **Bar soap, Liquid hand soap, Hand sanitizer** | **13.8** |
| **Agriculture/Forestry/Fisheries/Livestock** | | | |
| **pre** | | **post** | |
| **combination** | **%** | **combination** | **%** |
| **Bar soap** | **66.7** | **Bar soap** | **16.7** |
| **Liquid hand soap** | **0.0** | **Liquid hand soap** | **0.0** |
| **Bar soap, Liquid hand soap** | **33.3** | **Bar soap, Liquid hand soap** | **16.7** |
| **Bar soap, Hand sanitizer** | **0.0** | **Bar soap, Hand sanitizer** | **33.3** |
| **Liquid hand soap, Hand sanitizer** | **0.0** | **Liquid hand soap, Hand sanitizer** | **0.0** |
| **Bar soap, Liquid hand soap, Hand sanitizer** | **0.0** | **Bar soap, Liquid hand soap, Hand sanitizer** | **33.3** |
| **Housewife** | | | |
| **pre** | | **post** | |
| **combination** | **%** | **combination** | **%** |
| **Bar soap** | **62.8** | **Bar soap** | **8.3** |
| **Liquid hand soap** | **9.7** | **Liquid hand soap** | **4.8** |
| **Bar soap, Liquid hand soap** | **18.6** | **Bar soap, Liquid hand soap** | **3.4** |
| **Bar soap, Hand sanitizer** | **4.8** | **Bar soap, Hand sanitizer** | **39.3** |
| **Liquid hand soap, Hand sanitizer** | **2.1** | **Liquid hand soap, Hand sanitizer** | **16.6** |
| **Bar soap, Liquid hand soap, Hand sanitizer** | **2.1** | **Bar soap, Liquid hand soap, Hand sanitizer** | **27.6** |
| **Student** | | | |
| **pre** | | **post** | |
| **combination** | **%** | **combination** | **%** |
| **Bar soap** | **61.3** | **Bar soap** | **13.8** |
| **Liquid hand soap** | **2.5** | **Liquid hand soap** | **5.0** |
| **Bar soap, Liquid hand soap** | **17.5** | **Bar soap, Liquid hand soap** | **8.8** |
| **Bar soap, Hand sanitizer** | **11.3** | **Bar soap, Hand sanitizer** | **33.8** |
| **Liquid hand soap, Hand sanitizer** | **1.3** | **Liquid hand soap, Hand sanitizer** | **11.3** |
| **Bar soap, Liquid hand soap, Hand sanitizer** | **6.3** | **Bar soap, Liquid hand soap, Hand sanitizer** | **27.5** |
| **Freelancer** | | | |
| **pre** | | **post** | |
| **combination** | **%** | **combination** | **%** |
| **Bar soap** | **52.9** | **Bar soap** | **17.6** |
| **Liquid hand soap** | **5.9** | **Liquid hand soap** | **0.0** |
| **Bar soap, Liquid hand soap** | **17.6** | **Bar soap, Liquid hand soap** | **11.8** |
| **Bar soap, Hand sanitizer** | **11.8** | **Bar soap, Hand sanitizer** | **29.4** |
| **Liquid hand soap, Hand sanitizer** | **0.0** | **Liquid hand soap, Hand sanitizer** | **17.6** |
| **Bar soap, Liquid hand soap, Hand sanitizer** | **11.8** | **Bar soap, Liquid hand soap, Hand sanitizer** | **23.5** |
| **Religious** | | | |
| **pre** | | **post** | |
| **combination** | **%** | **combination** | **%** |
| **Bar soap** | **50.0** | **Bar soap** | **100.0** |
| **Liquid hand soap** | **0.0** | **Liquid hand soap** | **0.0** |
| **Bar soap, Liquid hand soap** | **0.0** | **Bar soap, Liquid hand soap** | **0.0** |
| **Bar soap, Hand sanitizer** | **50.0** | **Bar soap, Hand sanitizer** | **0.0** |
| **Liquid hand soap, Hand sanitizer** | **0.0** | **Liquid hand soap, Hand sanitizer** | **0.0** |
| **Bar soap, Liquid hand soap, Hand sanitizer** | **0.0** | **Bar soap, Liquid hand soap, Hand sanitizer** | **0.0** |
| **Soldier** | | | |
| **pre** | | **post** | |
| **combination** | **%** | **combination** | **%** |
| **Bar soap** | **33.3** | **Bar soap** | **33.3** |
| **Liquid hand soap** | **0.0** | **Liquid hand soap** | **0.0** |
| **Bar soap, Liquid hand soap** | **33.3** | **Bar soap, Liquid hand soap** | **0.0** |
| **Bar soap, Hand sanitizer** | **33.3** | **Bar soap, Hand sanitizer** | **0.0** |
| **Liquid hand soap, Hand sanitizer** | **0.0** | **Liquid hand soap, Hand sanitizer** | **0.0** |
| **Bar soap, Liquid hand soap, Hand sanitizer** | **0.0** | **Bar soap, Liquid hand soap, Hand sanitizer** | **66.7** |
| **Part-time** | | | |
| **pre** | | **post** | |
| **combination** | **%** | **combination** | **%** |
| **Bar soap** | **77.8** | **Bar soap** | **11.1** |
| **Liquid hand soap** | **0.0** | **Liquid hand soap** | **0.0** |
| **Bar soap, Liquid hand soap** | **11.1** | **Bar soap, Liquid hand soap** | **0.0** |
| **Bar soap, Hand sanitizer** | **11.1** | **Bar soap, Hand sanitizer** | **44.4** |
| **Liquid hand soap, Hand sanitizer** | **0.0** | **Liquid hand soap, Hand sanitizer** | **11.1** |
| **Bar soap, Liquid hand soap, Hand sanitizer** | **0.0** | **Bar soap, Liquid hand soap, Hand sanitizer** | **33.3** |
| **Unemployment/Leave** | | | |
| **pre** | | **post** | |
| **combination** | **%** | **combination** | **%** |
| **Bar soap** | **73.1** | **Bar soap** | **13.5** |
| **Liquid hand soap** | **5.8** | **Liquid hand soap** | **5.8** |
| **Bar soap, Liquid hand soap** | **7.7** | **Bar soap, Liquid hand soap** | **5.8** |
| **Bar soap, Hand sanitizer** | **5.8** | **Bar soap, Hand sanitizer** | **46.2** |
| **Liquid hand soap, Hand sanitizer** | **3.8** | **Liquid hand soap, Hand sanitizer** | **7.7** |
| **Bar soap, Liquid hand soap, Hand sanitizer** | **3.8** | **Bar soap, Liquid hand soap, Hand sanitizer** | **21.2** |

**Table S4** Comparison of average handwashing frequency and average handwashing duration pre- and post-COVID-19 by age.

| **Usage pattern** | **Frequency (N (SD))** | | **Time (Second (SD))** | |
| --- | --- | --- | --- | --- |
| **COVID-19** | **pre** | **post** | **pre** | **post** |
| **20s^***,###^** | **6.4 (4.3)** | **9.4 (7.9)** | **16.9 (15.8)** | **29.3 (41.5)** |
| **30s^***,###^** | **7.3 (5.4)** | **10.6 (7.8)** | **16.9 (13.8)** | **27.5 (30.6)** |
| **40s^***,##^** | **7.3 (4.4)** | **10.1 (6.1)** | **20.9 (25.6)** | **30.1 (37.1)** |
| **50s^***,###^** | **6.9 (4.2)** | **10.0 (6.3)** | **23.0 (31.6)** | **35.8 (41.6)** |
| **60s^**,##^** | **6.7 (4.2)** | **9.5 (9.0)** | **23.3 (27.1)** | **36.2 (42.2)** |

Significances of handwashing frequency between before and after COVID-19 were indicated as follows; *p-value<0.05, **p-value<0.01 and ***p-value<0.001. Significances of handwashing duration between before and after COVID-19 were indicated as follows; #p-value<0.05, ##p-value<0.01 and ###p-value<0.001.

**Table S5** Comparison of average handwashing frequency and average handwashing duration pre- and post-COVID-19 by occupation.

| **Usage pattern** | **Frequency (N (SD))** | | **Time (Second (SD))** | |
| --- | --- | --- | --- | --- |
| **COVID-19** | **pre** | **post** | **pre** | **post** |
| **Professionals^***,##^** | **7.2 (4.9)** | **11.3 (9.2)** | **20.1 (24.9)** | **32.8 (40.6)** |
| **Business/Management/Office^***,###^** | **6.8 (4.0)** | **9.4 (5.3)** | **18.3 (18.2)** | **28.1 (29.5)** |
| **Self-employment^##^** | **6.6 (3.8)** | **10.6 (10.8)** | **26.5 (41.9)** | **36.8 (43.8)** |
| **Sales/Service^**,##^** | **7.9 (5.8)** | **10.7 (7.9)** | **18.3 (17.9)** | **29.2 (29.9)** |
| **Production/Technical^**,#^** | **7.0 (4.7)** | **10.8 (7.8)** | **22.4 (21.8)** | **40.5 (55.2)** |
| **Agriculture/Forestry/Fisheries/Livestock** | **10.5 (14.8)** | **14.5 (18.0)** | **19.5 (10.4)** | **23.1 (19.3)** |
| **Housewife^***,#^** | **7.6 (4.7)** | **10.4 (6.9)** | **24.6 (31.9)** | **34.9 (38.5)** |
| **Student^**,#^** | **6.2 (3.6)** | **8.5 (5.1)** | **18.0 (18.5)** | **35.1 (60.1)** |
| **Freelancer** | **6.0 (2.5)** | **7.7 (3.7)** | **31.3 (35.3)** | **47.8 (54.8)** |
| **Religious** | **6.0 (1.4)** | **13.5 (9.2)** | **7.6 (6.5)** | **13.8 (15.3)** |
| **Soldier** | **4.3 (3.2)** | **5.7 (2.9)** | **20.7 (12.0)** | **29.5 (74.5)** |
| **Part-time^#^** | **7.2 (3.9)** | **8.9 (5.4)** | **8.4 (6.1)** | **18.5 (12.0)** |
| **Unemployment/Leave^*,#^** | **5.9 (4.4)** | **9.3 (8.5)** | **14.4 (11.5)** | **22.0 (22.5)** |

Significances of handwashing frequency between before and after COVID-19 were indicated as follows; *p-value<0.05, **p-value<0.01 and ***p-value<0.001. Significances of handwashing duration between before and after COVID-19 were indicated as follows; #p-value<0.05, ##p-value<0.01 and ###p-value<0.001.

**Table S6** Comparison of bar soap, liquid hand soap and hand sanitizer usage patterns of consumers using products pre- and post-COVID-19.

| **Usage pattern** | **Product** | **COVID-19** | **P5** | **P25** | **P50** | **P75** | **P95** | **Mean** | **SD** | **p-value** |
| --- | --- | --- | --- | --- | --- | --- | --- | --- | --- | --- |
| **Frequency** | **Bar soap (n)***** | **pre** | **1** | **3** | **5** | **6** | **10** | **5.08** | **3.60** | **3.49.E-05** |
|  |  | **post** | **1** | **3** | **5** | **7** | **15** | **5.94** | **4.97** |  |
|  | **Liquid hand soap (n)***** | **pre** | **1** | **2** | **3** | **5** | **10** | **3.88** | **3.25** | **1.87.E-16** |
|  |  | **post** | **1** | **2** | **5** | **7.75** | **15** | **5.85** | **5.57** |  |
|  | **Hand sanitizer (n)***** | **pre** | **0.1** | **0.3** | **2** | **3** | **7.75** | **2.37** | **3.52** | **0.005** |
|  |  | **post** | **0.3** | **1** | **3** | **5** | **10** | **3.25** | **3.11** |  |
| **Time** | **Bar soap (sec)***** | **pre** | **3** | **5** | **10** | **20** | **49** | **15.81** | **18.82** | **2.91.E-08** |
|  |  | **post** | **3** | **10** | **15** | **30** | **60** | **22.25** | **28.68** |  |
|  | **Liquid hand soap (sec)***** | **pre** | **5** | **10** | **20** | **30** | **67.5** | **29.45** | **32.66** | **1.09.E-06** |
|  |  | **post** | **8.45** | **20** | **30** | **40** | **120** | **39.52** | **44.74** |  |
|  | **Hand sanitizer (sec)*** | **pre** | **3** | **9.4** | **10** | **20** | **30** | **14.14** | **10.49** | **0.042** |
|  |  | **post** | **5** | **10** | **10** | **20** | **30.75** | **16.16** | **14.33** |  |
| **Amount** | **Bar soap (sec)***** | **pre** | **10** | **15** | **30** | **40** | **100** | **34.85** | **36.46** | **2.34.E-09** |
|  |  | **post** | **10** | **20** | **30** | **50** | **150** | **47.68** | **51.96** |  |
|  | **Liquid hand soap (pump)***** | **pre** | **1** | **1** | **2** | **2** | **3** | **1.71** | **0.80** | **1.32.E-08** |
|  |  | **post** | **1** | **1** | **2** | **2** | **3** | **1.98** | **0.94** |  |
|  | **Hand sanitizer (pump)*** | **pre** | **1** | **1** | **1** | **2** | **3** | **1.46** | **0.77** | **0.017** |
|  |  | **post** | **1** | **1** | **1** | **2** | **3** | **1.63** | **0.87** |  |

*p-value<0.05, ** p-value <0.01 and *** p-value <0.001
